# Indisulam Reduces Viability and Regulates Apoptotic Gene Expression in Pediatric High-Grade Glioma Cells

**DOI:** 10.3390/biomedicines11010068

**Published:** 2022-12-27

**Authors:** Caio C. D. Monção, Carlos A. Scrideli, Augusto F. Andrade, Mariano S. Viapiano, Carlos G. Carlotti, Daniel Antunes Moreno, Mirella Baroni, Luiz G. Tone, Silvia A. Teixeira

**Affiliations:** 1 Department of Paediatrics, Ribeirão Preto Medical School, University of São Paulo, Ribeirão Preto 14049-900, São Paulo, Brazil; 2 Department of Human Genetics, McGill University, Montreal, QC H3A 0C7, Canada; 3 Department of Neurosurgery, Brigham and Women’s Hospital and Harvard Medical School, Boston, MA 02115, USA; 4 Departments of Neurosurgery and Neuroscience & Physiology, SUNY Upstate Medical University, Syracuse, NY 13210, USA; 5 Department of Surgery and Anatomy, Ribeirão Preto Medical School, University of São Paulo, Ribeirão Preto 14049-900, São Paulo, Brazil; 6 Molecular Oncology Research Center, Barretos Cancer Hospital, Barretos 14784-400, São Paulo, Brazil; 7 Department of Genetics, Ribeirão Preto Medical School, University of São Paulo, Ribeirão Preto 14049-900, São Paulo, Brazil

**Keywords:** carbonic anhydrase, cell metabolism, hypoxia, apoptosis, microenvironment tumor, pediatric high-grade glioma

## Abstract

Pediatric high-grade glioma (pHGG) is one of the most aggressive brain tumors. Treatment includes surgery, radiotherapy, chemotherapy, or combination therapy in children older than 3–5 years of age. These devastating tumors are influenced by the hypoxic microenvironment that coordinatively increases the expression of carbonic anhydrases (CA9 and CA12) that are involved in pH regulation, metabolism, cell invasion, and resistance to therapy. The synthetic sulphonamide Indisulam is a potent inhibitor of CAs. The aim of this study was to evaluate the effects of Indisulam on CA9 and CA12 enzymes in pHGG cell lines. Our results indicated that, under hypoxia, the gene and protein expression of CA9 and CA12 are increased in pHGG cells. The functional effects of Indisulam on cell proliferation, clonogenic capacity, and apoptosis were measured in vitro. CA9 and CA12 gene and protein expression were analyzed by RT-PCR and western blot. The treatment with Indisulam significantly reduced cell proliferation (dose-time-dependent) and clonogenic capacity (*p* < 0.05) and potentiated the effect of apoptosis (*p* < 0.01). Indisulam promoted an imbalance in the anti-apoptotic BCL2 and pro-apoptotic BAX protein expression. Our results demonstrate that Indisulam contributes to apoptosis via imbalance of apoptotic proteins (BAX/BCL2) and suggests a potential to overcome chemotherapy resistance caused by the regulation these proteins.

## 1. Introduction

The Central Nervous System (CNS) tumors are the leading cause of cancer-related death in children [1] and exhibit molecular and genetic signatures different from those found in adult patients [2]. Pediatric high-grade glioma (pHGG) is a heterogeneous and aggressive primary brain tumor characterized by a rapid and infiltrative growth pattern [3,4]. Unfortunately, there is still no efficient treatment protocol for pHGG, which remains a devastating disease with significant morbidity and mortality [1,3,4]. The maximal safe surgical resection is recommended, and radiotherapy seems to be more effective for children over ten years old but not for younger patients [5]. Despite extensive research for novel treatment strategies, no new drug has dramatically increased the patient’s survival in the last decades, and the 5-year overall survival is nearly 17% [5,6]. HGGs are locally invasive and highly vascular tumors with extensive areas of necrosis and hypoxia. Hypoxia is a hallmark microenvironmental condition of HGG [7,8] that contributes the tumor development and growth [9]. In response to tumor growth, the microenvironment changes continuously and promotes specific conditions such as hypoxia and acidosis [10,11]. The tumor behavior is also affected by acidosis and, especially, by high lactate levels. In addition, studies described that acidosis promotes cell motility, migration, degradation, remodeling of the extra-cellular matrix (ECM,) and the radio and chemoresistance of tumor cells [10,11]. In this microenvironment, and under low oxygen concentrations, carbonic anhydrases (CAs) enzymes, are overexpressed and stimulate cell survival, invasion, and proliferation of tumor cells [12,13].

Carbonic Anhydrases (CAs) are zinc-dependent metalloenzymes that catalyze the reversible hydration of carbon dioxide to bicarbonate, being involved in acid-base balance and other physiological processes [14]. The transmembrane isozymes CA9 and CA12 are overexpressed in many tumor types and their overexpression is correlated with poor prognosis in high-grade gliomas [11,15,16]. In addition, CAs have been described as potential anti-cancer therapeutic targets [17,18] and compounds derived of sulfonamide, such as Indisulam, have shown antitumor effects in vitro and in vivo [19,20].

Indisulam/E7070 (N-(3-chloro-7-indolyl)-1,4-benzenedisulfonamide) is reported to be a potential inhibitor of CA9 and CA12 [21], which are overexpressed in diffuse gliomas from adult patients [20,22,23]. Studies have described that Indisulam targets multiple checkpoints of cell cycle phases, downregulates cyclins via p21/p53 dependent mechanisms, and promotes a significant impact on cellular metabolism [24,25,26]. Moreover, Indisulam can penetrate the blood-brain barrier [20,27] and may have promising anti-neoplastic effects in HGG. Despite this, the functions of CA9 and CA12 and the effects of Indisulam on pHGG are still unknown. In this study, we demonstrate that Indisulam reduces cellular growth and contributes to apoptosis via BCL and BAX imbalance.

## 2. Materials and Methods

### 2.1. Cell Culture

Pediatric HGG cell lines SF188 and KNS-42 were provided by Nada Jabado and Damien Faury, McGill University, Canada, and purchased from the Japanese Collection of Research Biosources Cell Bank—through the Rio de Janeiro Cell Bank, respectively. The cells were authenticated by short tandem repeat profiling, and mycoplasma testing was carried out. KNS-42 was cultured in MEM culture medium supplemented with 1% glutamine, 100 mg/mL streptomycin, 100 U/mL penicillin, and 5% fetal bovine serum (pH 7.2–7.4). SF188 was cultured in HAM F10 supplemented with 60 mg/L penicillin, 100 mg/mL streptomycin, and 10% (*v*/*v*) fetal bovine serum (Gibco, Life Technologies, Carlsbad, CA, USA).

### 2.2. Hypoxia Conditions—Treatment of Cells with Cobalt Chloride

To mimic hypoxic cultures, cells were cultured in the presence of Cobalt Chloride (CoCl_2_) (Mallinckrodt Chemicals, Dublin, Ireland). SF188 and KNS-42 cells were initially incubated with CoCl_2_ at increasing concentrations of 50, 100, and 150 μM for 24 h. For subsequent functional experiments, SF188 and KNS-42 were incubated with CoCl_2_ at 50 μM and 100 μM, respectively. 

### 2.3. Drug and Treatments

Indisulam (Medkoo Bioscience, Morrisville, NC, USA) was dissolved as stock at 100 mM in DMSO and stored at −20 °C until use according to manufacturer recommendations. 

### 2.4. Cell Viability Assay

A total of 2 × 10^3^ cells were seeded in quadruplicate in 96-well plates and maintained under standard culture conditions, under hypoxia for 24 h. The cells were treated with Indisulam at 2 to 256 μM and incubated for 24, 48, and 72 h under hypoxia. At each treatment interval, a resazurin (Sigma-Aldrich Co., St. Louis, MO, USA) solution was added to the plates (10% of the initial volume). The plates were incubated for 4 h and read at 570 nm (iMax Microplate Reader (Bio-Rad, Hercules, CA, USA). These data were used to obtain the IC_50_ value for KNS-42 and SF188, defined as the concentrations necessary for 50% cell viability reduction, using the Calcusyn software (Biosoft, Ferguson, MO, USA). Three independent experiments were performed in quadruplicate. The results are expressed as mean plus standard deviation and were compared between treatments and control.

### 2.5. Clonogenic Survival 

To carry out the clonogenic assay, 500 cells were seeded in triplicate in six-well plates. After 24 h incubation with CoCl_2_, the cells were treated with Indisulam (2 to 256 µM) for 48 h. Thus, these tests allowed us to identify the lowest dose of Indisulam required to inhibit clonogenicity. The cells were washed with PBS, and a culture medium without the drug was added to permit colony growth for approximately 9–11 days until the colonies were visible but not confluent. Colonies were fixed in methanol and stained with 1% Giemsa. Colonies of at least 50 cells were counted with a magnifying glass. The plating efficacy (PE) represents the percentage of cells seeded that grow into colonies under a specific culture condition of a given cell line. It was calculated as described by Franken et al. [28]. Three independent experiments were performed.

### 2.6. Apoptosis Assay 

The assay for cell death detection was carried out by labeling apoptotic cells with annexin V fluorescein isothiocyanate (BD Biosciences, San Jose, CA, USA) and necrotic cells with propidium iodide (PI). To perform the experiments, 4 × 10^4^ KNS-42 or SF188 cells were incubated with CoCl_2_ (100 and 50 μL, respectively) for 24 h. After cells were treated with the different concentrations of Indisulam (8, 32, 128, and 256 µM) for 48 h, they were trypsinized, washed with ice-cold PBS, and resuspended in 1X annexin binding buffer (BD Biosciences, San Jose, CA, USA). The cells were labeled with annexin V and propidium iodide (PI) solution and analyzed using a BD FACSCalibur™ flow cytometer (BD Biosciences, San Jose, CA, USA). The values represent the mean and standard deviation of three independent experiments performed in triplicate.

### 2.7. mRNA Extraction and RT-qPCR

To evaluate the effect of Indisulam on *CA9* and *CA12* gene expression (refs. Hs00154208 and Hs01080909), the cells were exposed to CoCl_2_ for 24 h and treated with Indisulam (169 µM) for 48 h. mRNA was extracted using the Trizol^®^ reagent (Invitrogen Inc., Carsdab, CA, USA) according to the manufacturer’s instructions. cDNA was synthesized using High Capacity^®^ kit (Applied Biosystems, Foster City, CA, USA). qRT-PCR was performed using the QuantStudio 12k Flex device (Applied Biosystems, Foster City, CA, USA). Samples treated with vehicle were used as controls. TBP (TATA binding protein, ref. 4326322E) and HPRT (hypoxanthine guanine phosphoribosyl transferase, ref. 4326321E) genes were used as internal mRNA controls. The relative quantification of gene expression was determined using the 2^−ΔΔCT^ method [29].

### 2.8. Protein Extraction and Western Blotting

To evaluate the effect of Indisulam on protein expression of HIF1α, CA9, CA12, BAX, and BCL-2, the cells were exposed to CoCl_2_ for 24 h, subjected to treatment with the drug (169 µM) for 48 h, and then collected. Protein extraction was performed using RIPA lysis buffer (Sigma Aldrich Co., Saint Louis, MO, USA) with protease and phosphatase inhibitors according to manufacturer’s guidance. Protein concentration was determined by Bradford’s method [30]. Subsequently, 40 µg of the samples were separated by SDS-PAGE electrophoresis. The proteins were transferred to nitrocellulose membranes and then incubated in a 5% non-fat milk solution in 0.1% TBST for 2 h at room temperature. Following this, the membranes were incubated overnight with specific primary antibodies for each protein, diluted according to the manufacturers’ instructions (Table 1). The levels of the GAPDH protein and β-actin were used as an endogenous control in all experiments. The membranes were then washed with TBST and incubated with specific peroxidase-conjugated secondary antibody (Santa Cruz Biotechnology, Santa Cruz, CA, USA) for 1 h and submitted to another wash cycle. The protein bands were visualized using the ECL™ Prime Western Blotting Detection Reagent (GE Healthcare, Amersham, UK) and ChemiDoc system (Bio-Rad Laboratories, Hercules, CA, USA). The membranes were exposed for 10 to 500 s and then analyzed. The relative quantification of protein expression was determined using ImageJ software (National Institutes of Health, Bethesda, MD, USA).

### 2.9. Statistical Analysis

Results are presented as mean with standard deviation. Significant differences were tested using Student’s t test for gene and protein expression, while one-way analysis of variance (ANOVA) with a post-hoc Bonferroni test was carried out for the remaining experiments. All analyses were performed using SPSS 20.0 software (SPSS, Chicago, IL, USA) or Prism GraphPad6.0 software (Graph Pad, San Diego, CA, USA) with level of significance *p* < 0.05. 

## 3. Results

### 3.1. CoCl_2_ Induces Hypoxia-like Condition in pHGG Cell Lines

**Figure 1:**
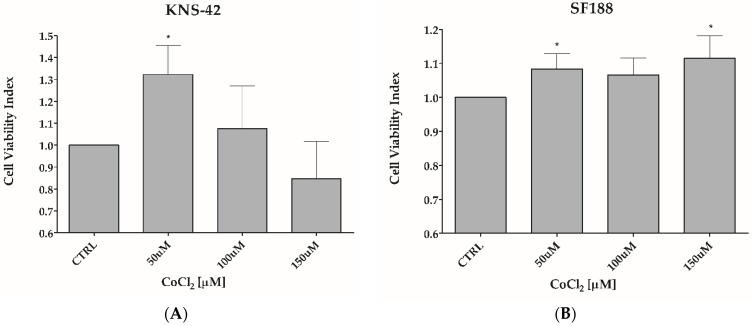
CoCl_2_ treatment did not reduce the cell viability of pHGG cell lines. Analysis of cell viability of KNS-42 and SF188 cell lines when treated with different concentrations of CoCl_2_ (50, 100, and 150 µM) for 24 h. Viability was measured using the Resazurin method. * *p* < 0.05.

**Figure 2:**
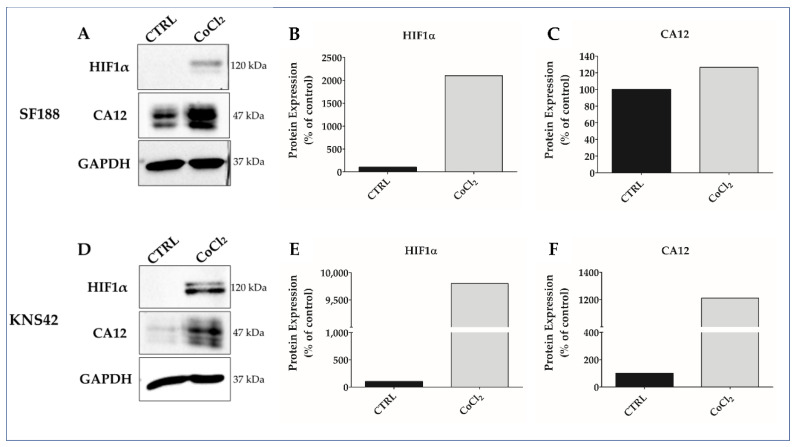
CoCl_2_ increases protein expression of HIF1α and CA12 in SF188 and KNS-42 pHGG cell lines. The HIF1α and CA12 protein expression was evaluated by Western blotting in SF188 (**A**) and KNS-42 (**D**) cell lines. Graph bars indicate that the treatment for 24 h with CoCl_2_ 50 μM (SF188) and 100 μM (KNS-42) increased the expression of HIF1α (**B**,**E**) and CA12 (**C**,**F**) compared to the control (CTRL). GAPDH was the endogenous protein used. Relative quantification of protein expression was determined using the ImageJ software.

Hypoxia mimicry was performed by treating SF188 and KNS-42 cells with 50 μM, 100 μM, or 150 μM CoCl_2_, respectively, for 24 h (Figure 1). The treatment with 50 μM or 100 μM CoCl_2_, did not reduce cell viability (Figure 1A,B) but drastically upregulated HIF1α expression for SF188 (Figure 2A,B) and KNS-42 cells (Figure 2D,E), as well as CA12 pro-tein expression for KNS-42 cells (Figure 2D,F), in addition to positively modulating (30%) CA12 protein expression in SF188 cells (Figure 2A,C). It is essential to point out that higher doses (such as 150 μM of CoCl_2_ for KNS-42 cells) can reduce cell viability, indicating a cytotoxic effect at concentrations above the dose selected for the experiments in this study (Figure 1A).

### 3.2. Indisulam Decreases Viability in pHGG Cells

**Figure 3:**
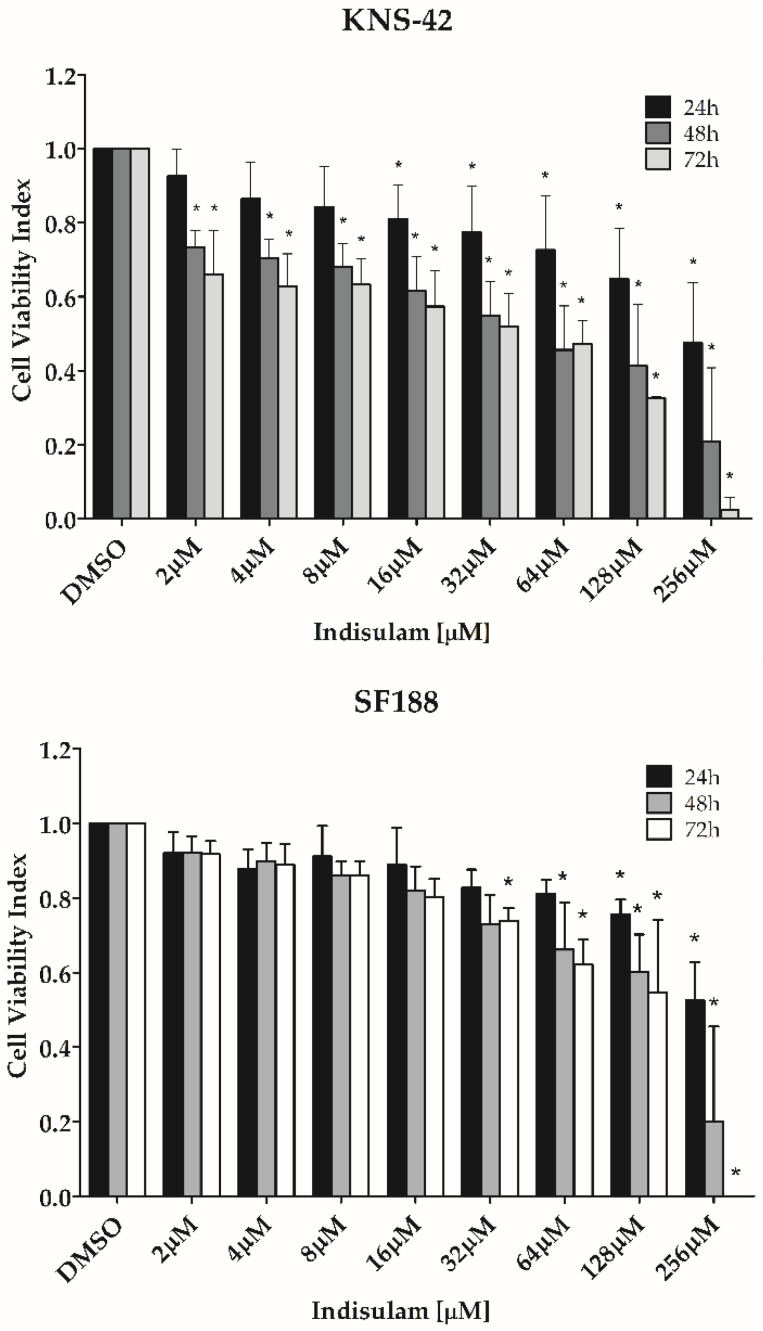
Indisulam decreases cell viability in pHGG cell lines. To chemically induce hypoxia, SF188 and KNS-42 cell lines were pre-treated with CoCl_2_ (50 μM and 100 μM, respectively) for 24 h. After hypoxia induction, the cells were treated with increasing concentrations of Indisulam (0–256 μM) for 24 to 72 h. The cell viability was quantified by the resazurin method. The plot shows the mean ± standard deviation of three independent experiments. * *p* < 0.05 indicates a significant difference between the treated cells and the DMSO vehicle control.

Because CA9 and CA12, targets of Indisulam, were increased in hypoxia, the subsequent effects of this drug were tested under the same hypoxia-like conditions. Cell viability assays demonstrated that Indisulam decreased the viability of both pHGG cell lines in a dose-dependent manner, showing that KNS-42 is more sensitive to Indisulam treatment than SF188 (Figure 3).

### 3.3. Indisulam Reduces Clonogenicity and Increased Apoptosis in pHGG Cell Line

**Figure 4:**
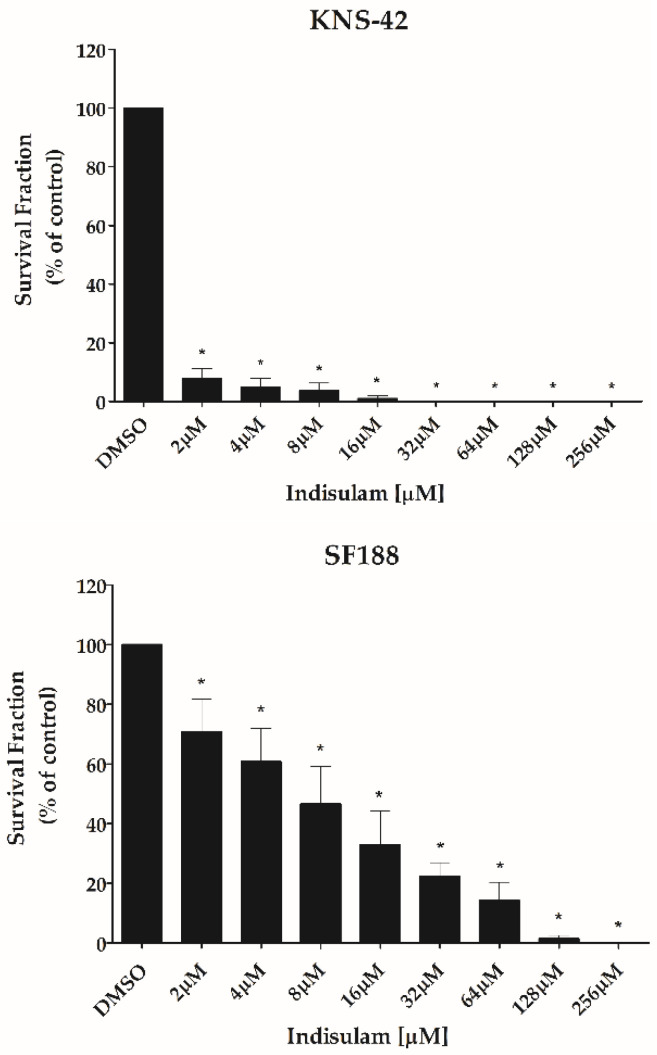
Indisulam reduces the clonogenic capacity of pHGG cell lines. SF188 and KNS-42 cells were pre-incubated in hypoxia-mimic conditions (CoCl_2_ at 50 μM and 100 μM, respectively) for 24 h. The cells were treated with Indisulam for 48 h and further cultured for 9 to 11 days to assess colony formation. Cells treated with vehicle alone (DMSO) served as controls. The colonies formed were normalized to the plating efficiency (FP) and the survival fraction obtained was compared to the control (DMSO). * *p* < 0.001 indicates a significant difference between the treated cells and the control.

**Figure 5:**
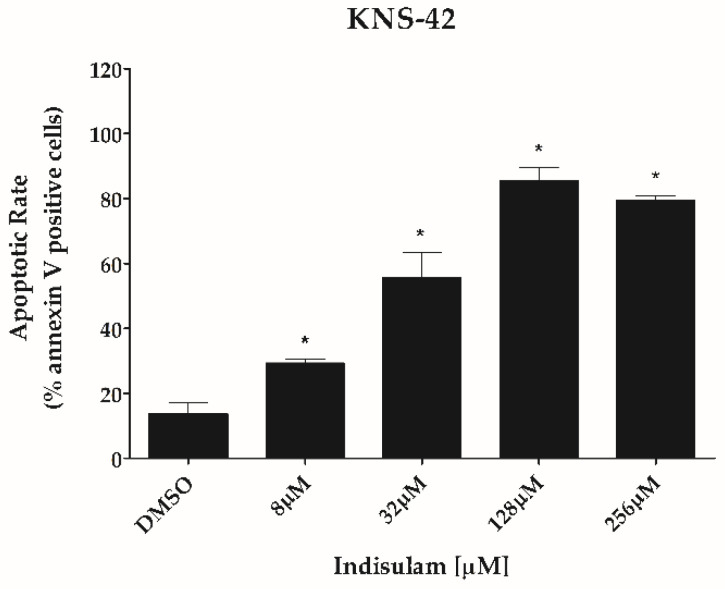

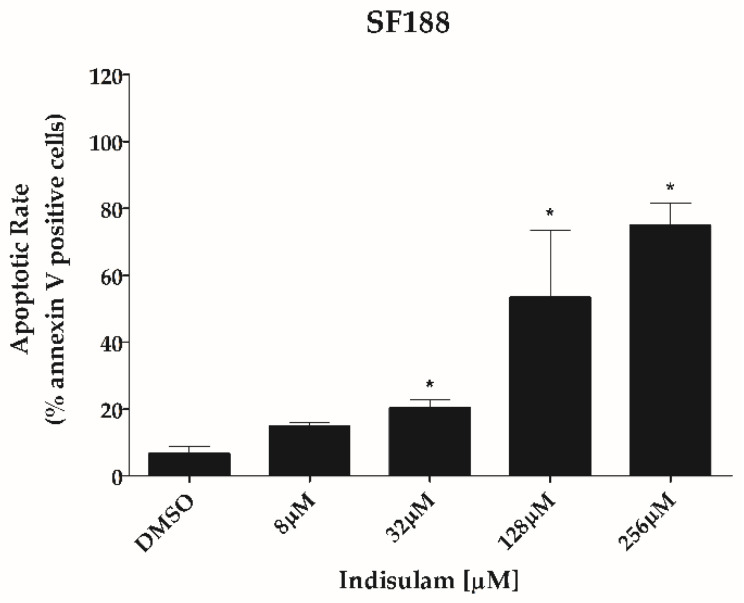
Indisulam induces apoptosis in pHGG cell lines. To induce hypoxia, the cells SF188 and KNS-42 were incubated for 24 h with CoCl_2_ (50 μM and 100 μM, respectively) and then treated with different concentrations of Indisulam (0–256 μM) for 48 h, as indicated on bar graphs. Cell death by apoptosis was assessed by annexin-V and propidium iodide and analyzed by flow cytometry. * *p* < 0.05 indicates a significant difference between the treated cells and the DMSO control.

Indisulam significantly decreased the ability of both KNS-42 and SF188 cells to form colonies at all doses tested when compared to the control group (*p* < 0.001). As observed in the viability assays, the sensitivity of KNS-42 to Indisulam was higher than SF188 (Figure 4). Total inhibition of clonogenicity was observed at an Indisulam dose of 32 μM for KNS-42 and 256 μM for SF188 cells (Figure 4). Similarly, Indisulam increased apoptosis rates determined by annexin-V/propidium iodide staining (*p* < 0.05) (Figure 5). The cell apoptosis rate reached approximately 75% at the highest dose tested for both cell lines.

### 3.4. Indisulam Modulates CA9 and CA12 Gene and Protein Expression in pHGG Cells

**Figure 6:**
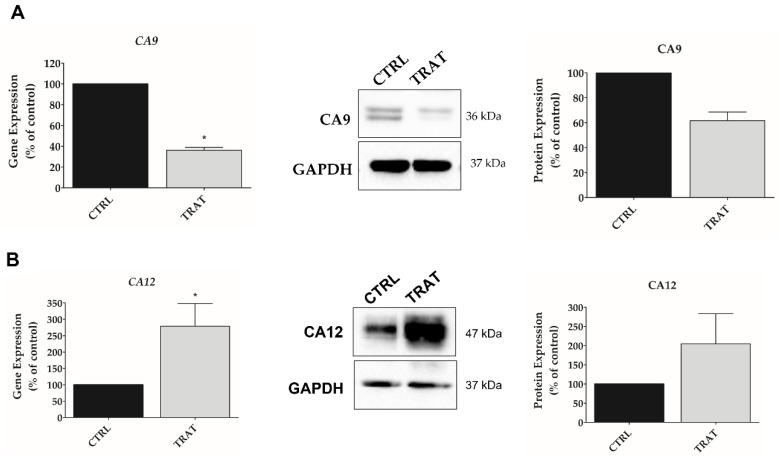
Indisulam decreases CA9 and increases CA12 expression in pHGG cells under hypoxia. SF188 cell line was incubated for 24 h under mimetic hypoxia condition (CoCl_2_ 50 μM) and treated with Indisulam (IC_50_) for 48 h. Gene and protein expression of CA9 (**A**) and CA12 (**B**) were measured by qRT-PCR and Western blotting, respectively. The bar graphs and Western blot image indicate that, the treatment of SF188 cells with Indisulam reduces CA9 gene and protein expression, while increasing the expression of CA12 compared to control. CTRL: control; TRAT: treatment with the Indisulam; GAPDH was used as loading control; quantification of relative expression was determined using ImageJ software. * *p* < 0.05.

The CA9 and CA12 expression were analyzed in SF188 cells incubated under hypoxia for 48 h in the presence or absence of Indisulam (applied at its IC_50_ dose). The treatment resulted in downregulation of CA9 expression at the mRNA and protein levels. However, Indisulam upregulated CA12 mRNA and protein expression (Figure 6A,B), suggesting a possible compensating effect parallel to the downregulation of CA9.

### 3.5. Indisulam Promotes Alteration in BAX and BCL-2 Protein Expression in pHGG Cells

**Figure 7:**
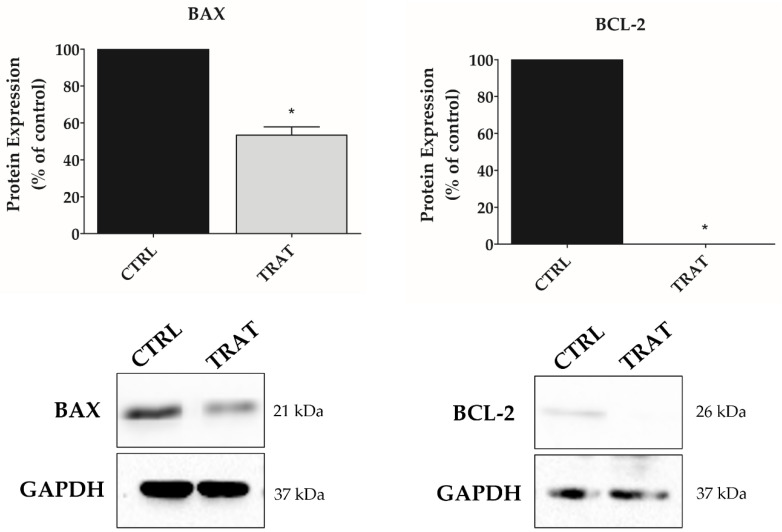
Indisulam alters the protein expression of BAX and BCL-2 in the pHGG cell line. SF188 cell was incubated under hypoxic conditions (CoCl_2_ 50 μM) for 24 h and treated with Indisulam (IC_50_), or vehicle, for 48 h, and then BAX and BCL-2 protein expression was analyzed by Western blotting. In the analysis, the relative quantification was determined using ImageJ software. Relative values were compared to the control. * *p* < 0.05 indicates a significant difference between the BAX protein expression of the treated cells and the DMSO control, whereas * *p* < 0.01 indicates a significant difference between the BCL-2 expression of the treated cells and the control. GAPDH was used as endogen control. CTRL: control; TRAT: treatment with Indisulam.

To understand the molecular changes underlying the apoptotic process, the expression of pro-apoptotic (BAX) and anti-apoptotic (BCL-2) factors was analyzed after treatment of SF188 cells with Indisulam under hypoxia (Figure 7). The results showed that BAX expression was decreased by 47% (*p* < 0.05), while BCL-2 expression was almost completely abolished when compared to the control (*p* < 0.01). Consequently, the BAX/BCL-2 ratio was increased in the group of cells treated with Indisulam when compared to the control.

## 4. Discussion

Pediatric high-grade gliomas represent a group of tumors with wide heterogeneity, as demonstrated by identifying subgroups with different genetic, epigenetic, and morphological characteristics [31]. Based on their molecular analysis, the WHO divides pediatric-type diffuse high-grade gliomas into four subgroups: Diffuse midline glioma H3 K27-altered, Diffuse hemispheric glioma H3 G34-mutant, Diffuse pediatric-type high-grade glioma H3-wildtype and IDH-wildtype and Infant-type hemispheric glioma [32]. Using information from the methylation profile of these tumors, as well as tumor location and altered oncogenic pathways, the German Cancer Research Center establishes six different subtypes: K27, G34, IDH, RTK-I, Mesenchymal and PXA-like [33]. Regarding the cells selected to realize this study, KNS-42 presents mutation in histone H3.3 G34V and activation of NMYC and IGFR1 pathways [34]. Among the pHGGs, histone H3 mutation is present in 40% of the total, being related to a less aggressive subtype with a better prognosis [35]. Already, SF188 has overexpression of PDGFRA and amplified MYC [34], the latter being present in only 8 to 9% of cases of pHGGs, being related to a poor prognosis [35]. However, targeted therapies for each subgroup of pediatric CNS tumors have not yet been established. Therefore, clinical treatments are generally based on traditional protocols, which lack long-term efficacy because they do not take into account the distinct characteristics of pHGG subgroups or their differences with adult tumors [36,37]. In addition to tumor heterogeneity, the presence of diffuse and ill-defined tumor borders [37], as well as hypoxic regions [12], reduce the efficacy of surgical resection [3,38] and adjuvant chemo- and radiotherapy [39]. In particular, the relationship between hypoxia and radioresistance of cancer has been demonstrated since the 1950s [40,41]. Under the hypoxic microenvironment of the tumor, CA9 and CA12 have an important role in the pH regulation and therefore contribute to treatment resistance and tumor progression [14,15,16,19,42]. Indeed, prior studies from our group have demonstrated that the inhibition of CA9 and CA12 can block cell cycle progression and sensitize adult GBM cells to the treatment of radio- (in vitro) and chemotherapy (in vitro and in vivo) [20]. However, to the best of our knowledge, the effect of anti-CA treatment on pediatric brain tumors has not been studied. 

In the present study, we have analyzed whether the treatment of pHGG with Indisulam has inhibitory effects on CA isozymes and cell viability, as previously shown with adult GBM cells. To induce hypoxia, we selected CoCl_2_, a chemical hypoxia model that increases HIF1α and upregulated genes that encode proteins related to pH regulation such as CA9 and CA12. Then, our results have shown that a hypoxia-mimic is sufficient to increase the expression of HIF1α, CA9, and CA12, suggesting that this is an adequate condition to investigate Indisulam’s effects on pHGG cells. 

Carbonic anhydrases, controlled by oxygen levels via the HIF1α [43], are overexpressed in several solid tumors, with a fundamental role in tumor pH homeostasis and very low, or negligible expression, in normal tissues [14]. The CoCl_2_ increases HIF1α in dose (25 µM to 200 µM) and time-dependent, according to the cell type selected, and generally, the stabilization is observed for 2 h, with a maximum of 12–48 h [44,45,46,47,48,49]. A similar effect of HIF expression was observed in breast cancer and HGG cells exposed to hypoxia conditions [34,44]. For the U87 GBM cell, in the treatment with CoCl_2_ 100 mM, the expression of HIF1α was time-dependent. In addition, doses ≤200 µM CoCl_2_, and 24 h of treatment, did not increase or decrease cell viability of glioblastoma and colon cancer; however, the viability decreased after 48 h of treatment [45,47]. In this way, recent publications have demonstrated that the hypoxia exposition for 72 h to 168 h significantly decreased the tumor cell viability [34,46,47]. According to Rana et al., the cytotoxic effect of CoCl_2_ is due to the inhibition of DNA repair pathways and accumulation of ROS (Reactive Oxygen Species), with consequent cell death [44]. In vitro, this cytotoxicity appears to be dose-dependent, as observed in breast cancer cells [44,49] and the KNS-42 cell line in the present study. Furthermore, using 50 and 100 µM of CoCl_2_ promoted an increase in the number of both cell lines selected to develop this study (SF188 and KNS-42). Thus, as the observed effect did not decrease but increased cell proliferation for the selected CoCl_2_ doses, the analysis of the Indisulam effect in the present work was not impaired. 

High expression of CAs, associated with high proliferation and expansion of tumor cells that deprive the tissues of oxygen, has been described in brain tumors and functionally linked to the malignant behavior of these cancer cells [50]. A metabolic adaptation of GBM to these oxygen concentrations is also present through the Warburg effect, leading to lactate production as a metabolic by-product and increasing resistance to radiotherapy [50]. Furthermore, malignant cells, especially tumor stem cells, can endure hypoxic conditions by changing their expression of genes involved in cell proliferation, metabolism, apoptosis, and angiogenesis, thus resulting in tumor expansion, treatment resistance, and metabolic adaptation [51]. The high expression of CA9 and CA12 in a hypoxic microenvironment is currently considered a potential prognostic biomarker and therapeutic target [10]. CAs have restricted expression in non-neoplastic tissues, suggesting that it may be possible to inhibit their pro-tumoral effects in the microenvironmental of the tumor, with minimal toxicity on normal tissues [52]. Accordingly, this study aimed to use Indisulam to inhibit the upregulation of CA9 and CA12 observed in hypoxic pHGG cells. Interestingly, we observed inhibition of mRNA and protein expression for CA9 but not for CA12. Similar results have been reported in adult GBM cells [20]. According to Chiche et al., 2009 [53], this effect might represent a compensatory feedback mechanism for tumor cells to maintain an intracellular alkaline pH.

Overall, our results suggest that Indisulam triggered an antitumor effect through the reduction of cell proliferation and clonogenicity of pHGG cells. The antitumor activity of Indisulam has been demonstrated in other tumor types [20,21,25,54,55]. This effect may be related to the ability of Indisulam to inhibit cell cycle progression through G1/S and G2/S phase disarray and decreased expression of cyclins A, B1, and E, and CDK2/CDK4 and altered glucose metabolism [56,57]. In turn, the antiproliferative effect of Indisulam may be caused by dysregulation of cellular pH after inhibition of CA9 [53,58,59,60,61], making the tumor more sensitive to external agents [11,62,63,64,65]. Inhibition of CAs by Indisulam has been shown to downregulate genes involved in chemoresistance and reduce proliferation capacity and invasion of cancer cells [21]. Then, a parallel mechanism by which hypoxia promotes pHGG chemoresistance is through inhibiting pro-apoptotic pathways. Hypoxia is considered a tumor microenvironment factor that favors the resistance to therapy. For instance, under hypoxic conditions, the proapoptotic protein appears to be modified and unable to interfere with pro-survival factors; therefore, an increase in the expression of antiapoptotic proteins has been reported [47,48]. For breast cancer, hypoxia upregulated proapoptotic protein expression [44]. In this way, the family of apoptosis-related proteins BCL-2 (B-cell lymphoma 2) inhibits apoptosis, and BAX (BCL-2-associated X protein) promotes a proapoptotic effect [47]. However, the criterion for programmed cell death is the imbalance in the ratio of BCL-2/BAX expression.

Our results indicate that Indisulam, inhibiting CA9, promotes apoptosis in pHGG cells in hypoxia. The BAX/BCL-2 ratio increase in the Indisulam-treated group makes evident the imbalance in the pro-survival machinery and triggering of the apoptotic process [66,67]. In this sense, the drastic reduction of the anti-apoptotic factor BCL-2, more markedly than the proapoptotic factor BAX, leads to cell death through the intrinsic pathway of apoptosis, which is controlled by members of the BCL-2 family [67,68]. The CA9 inhibition-induced apoptosis by other agents has also been described in colorectal [69], cervical [70,71] and breast cancer [72]. Inhibition of CA9 decreases intracellular pH, causing DNA damage and ROS accumulation that activate apoptosis [73]. Cianchi et al. have also found that the inhibition of CA9 leads to the phosphorylation of p38/MAPK as well as the synthesis of peroxynitrite [73]. In addition, the p38/MAPK factor is described as a mediator of apoptosis [74], possibly relating this pathway to apoptotic induction promoted by the inhibition of CAs. Recently, it was observed that the induction of apoptosis after inhibition of CA9 and CA12 is mediated by the activation of caspase 7, with an increase in ROS and a decrease in PARP-1 levels [75,76]. Moreover, Indisulam promotes the overexpression of p53 and p21, leading to apoptosis [25,77]. The pro-apoptotic stimulus is one of the most efficient effects in non-surgical treatments [78], and the fact that Indisulam presents this activity in pHGG cells highlights its potential in anti-cancer therapeutic strategies. 

The current standard-of-care chemotherapy to treat high-grade glioma, including pHGG, is Temozolomide (TMZ). However, the treatment is not curative, even with a combination of surgery, radiotherapy, and adjuvant chemotherapy, with relapse being a frequent outcome [79]. The best-known resistance mechanism to TMZ is effectuated by O6-Methylguanine-DNA methyltransferase (MGMT), which corrects DNA damage caused by TMZ and reduces the effectiveness of the treatment [80]. This resistance mechanism is probably also associated with the poor response to TMZ in pHGG [81,82]. The combination of TMZ with carbonic anhydrase inhibitors [52], including Indisulam [20], results in increased TMZ efficacy and indicates a promising strategy toward more effective treatments. In addition, Indisulam has already been tested in combination with another chemo- and radiotherapeutic agents in vitro, in vivo, and clinical studies, showing promising results as an adjuvant strategy [20,21,83,84,85]. Despite the limitation of working with established cell lines and in vitro models, several studies and different research groups with the purpose of better understanding the tumor biology of pediatric glioblastoma have already used the SF188 and KNS-42 cell lines, being two reliable models of investigation of this tumor subtype. Although in vitro tumor models with cell culture have provided essential tools for cancer research and serve as low-cost models for drug therapy investigation, serial passage of cell lines can further cause genotypic and phenotypic variation over an extended period. Therefore, further assays with different tumor models are needed to validate the results found in this study.

## 5. Conclusions

Indisulam demonstrated anti-neoplastic effects on pHGG cell lines by reducing proliferation and inducing apoptosis through the modulation of the BAX and BCL-2 balance. This regulation may indicate a mechanism for overcoming pHGG chemoresistance, encouraging further studies. Future work may confirm Indisulam as a promising adjuvant strategy associated with current conventional treatments for pHGG.

## Figures and Tables

**Table 1:** Primary antibodies for Western blotting analysis.

Antibody	Reference	Dilution
HIF1α	610958-BD	1:500
CA9	D10C10	1:1000
CA12	D75C6	1:1000
BAX	sc-493	1:250
BCL-2	sc-7382	1:1000
GAPDH	sc-47724	1:1000
β-actin	sc-47778	1:1000

## Data Availability

The original contributions presented in the study are included in the article. Further inquiries can be directed to the corresponding author.
